# On the Use of Nitric Acid Issues in Chronic Diseases

**Published:** 1847-02

**Authors:** P. A. Allaire

**Affiliations:** Aurora, Kane Co., Ill.


ARTICLE VI.

*On the use of Nitric Acid Issues in Chronic Diseases.* By P. A. Allaire, M.D.

We find, on consulting dictionaries, dispensatories, &c., nitric acid noticed chiefly as a tonic, alterative, and disinfectant. As a caustic, it is scarcely mentioned, although it possesses powers in this respect, inferior to no other article. As a cautery, for the formation of issues, I know of no authority recommending it; yet when used in its concentrated state, it seems to me much superior to the article usually had recourse to, viz. Potassa; for it will destroy the cutis down to the cellular tissue in from five to eight minutes, whereas the potassa requires nearly as many hours.

Metal at a white heat is alone more rapid in destroying the living tissue, but the application of a hot iron will not, even in idea, be tolerated by many patients, and, therefore, cannot be used. And, as quickness in the formation of issues, as well as in every other painful operation, is important, (the amount of pain being always proportionate to the time occupied) therefore the nitric acid, for this reason alone, may well merit attention. It is also as manageable as any other article, is very cleanly, and has nothing in its appearance terrible to the patient.

I wish here to notice the effect which late standard authorities attribute to issues. If I am correct, they are regarded chiefly as *revellants*, their utility being supposed to be in a ratio to the irritation they produce; little importance being assigned to the discharge. They are therefore made small, and irritation kept up in them by the use of issue peas and other foreign substances. Many of the older surgeons used the issue for the discharge, and yet, strangely enough, made them quite small. My experience has led me to use the issue as a drain, or as a means of steadily depleting a part; believing depletion as important in chronic inflammation as in acute, and that its activity should ever be proportioned to the activity of the disease. I believe, therefore, that the true method is to make the issues large, and in number varied as the disease under treatment is more or less extensive. It is also proper to avoid introducing into them any foreign substance, which always creates an unhealthy and offensive discharge, but, by a simple or slightly stimulative dressing, keep them healthy, allowing the healing process to go on as it will. By this mode of management the sore will generally close in twenty-five or thirty days, which I think a great advantage; because the constitution cannot in so short a time *adopt the discharge as a part of the healthy condition;* for the moment this occurs, then ceases the benefit of the issue as a drain, and also as a revellant. After the issues are healed, they may be again opened after the lapse of eight or ten days, if the continuance of the disease demand. And thus the depletion is again commenced, and kept up intermittingly as long as the particular case may require.

So successful have I found this method, that, in curable chronic diseases of an inflammatory or sub-inflammatory character, of some six and eight years continuance, it has, in my hands, very rarely failed of securing a favorable result and often has been successful without attention to diet, exercise, &c. I do not, however, underrate the value of revellants, or counter-irritants; as a class they are most important; yet they often fail in relieving long standing disease, even when perseveringly applied, and combined with constitutional treatment. Especially is this the case, when the difficulty is of an inflammatory or sub-inflammatory character. Under such circumstances, a large and freely suppurating surface, established as near the afflicted part as possible, unloads the turgid and enfeebled vessels, and enables them to resume a more healthy action. The following cases selected from many others, will serve to illustrate this method of using issues, and demonstrate their usefulness as a means of depletion.

*Case* 1st. D. H., a man of robust constitution, temperate habits, and general good health, æt. 33, called on me in June 1842, to prescribe for the following symptoms,—constant and excessive headache, with a feeling of fullness, face not flushed nor the veins distended; on stooping, dizziness and tinnitus aurium common, so that he has immediately to resume the upright position for fear of falling. The disease has been gradually increasing during the last five years in its severity, in consequence of which he has been compelled to relinquish his trade, that of an iron moulder. The usual remedies have been had recourse to, viz. bleeding, general and local; blisters, purgatives, &c., with scarcely temporary relief. I advised a large issue to be made on the nape of the neck, which after some persuasion was consented to. The issue I made of an oval form nearly two inches long by one and a quarter wide. No other remedy was used, and no particular attention given to diet. In six days the slough separated, and the discharge was profuse, which continued for twenty days, when it gradually healed, leaving the head free from every uncomfortable feeling. There has been no return of the disease to this time, and my old patient has long since resumed his former occupation as a moulder.

*Case* 2d. M. S. came under my care in Jan. last. She is a tall well formed woman aged about 30 years, mother of three children: the youngest four years old. Has been an invalid for some years, but worse since the birth of her last child. The symptoms have been all this time much as they now are, only as they have varied in severity.

*Symptoms.*—Costiveness, want of appetite, frequent nausea and vomiting, irregular pains in the abdomen, slight leucorrhœa, menses irregular, and deficient as to quantity. The spinal column is tender on pressure from the sixth cervical to the last lumbar vertebræ; the third and fourth dorsal are particularly tender. The woman looks pale and feeble, but is yet able to sit up and can sew for a short time. During the long period of her sufferings she has been subjected to a variety of treatment, sometimes with temporary benefit. She has had no issue or seton inserted, but has used ungt. tart. ant., with blisters to the spine, and a host of internal remedies.

*Treatment.*—Jan. 15th. Three issues were made along the sides of the spinal column, one opposite the third dorsal, one opposite the twelfth dorsal, and one opposite the fifth lumbar vertebræ. The issues were each about two inches long and one and a half wide at the broadest part, and of an oval form. The following pills were exhibited, as a tonic, and to relieve costiveness. ℞, ext. colocynth. comp. ʒj. Ferri sulph. ℈j in pill. xx. One of the above to be taken morning and night. During the treatment some attention was given to diet, but her other habits remained the same. Jan. 25th, the sloughs of the issues have separated and the discharge is profuse, indeed the debility is increased thereby. The bowels move kindly now with one pill per day. The appetite is much improved, the stomach digesting easily the lighter articles of food. No diminution of spinal irritation. Directed ℥j of infus. gentian three times daily, and the pills to be continued, one per day.

Feb. 12th. Spinal tenderness very much diminished, appetite improving, bowels regular with one pill every other night, patient's look much improved, but her strength is not increased. Issues yet discharge freely, but are beginning to heal.

℞, carb. ferri gr. viij, three times a day, and discontinued infus. gent. and pills.

Feb. 25th. Issues nearly healed; spinal tenderness gone; strength much improved; all uneasiness and pain of abdominal organs gone; stomach digests ordinary food with facility; uterine function natural and leucorrhœa has ceased. Directed the patient to continue the use of the carb. ferri for two or three weeks twice per day. No further treatment. She has not since had any return of her old difficulties.

*Case* 3d. S. H., a young man of 23 years of age, has been in feeble health for a period of four years, occasionally tolerably comfortable for two or three weeks, and then suffering so severely as to require active treatment.

*Symptoms.*—Frequent desire to pass urine, which he says is often bloody, always albuminous when I have tested it, pain in the loins, particularly on the left side; frequent griping pains in the bowels and often mucus or bloody discharges from them. These symptoms are constant, and occasionally of such severity as to call for general bleeding, with large doses of opiates. The vertebræ are not tender under pressure. The constant use of opium gives Mr. H. the only respite which he has from his disease. He is pale and much enfeebled, but is yet able to be "around." As usual in chronic cases this patient has tried everything and everybody, but with no more benefit than to relieve the exacerbations. I have treated him for two months with various internal remedies, and blisters and other revellants externally, without apparent benefit.

June 22d, 1846. Inserted two nitric acid issues, one over the left kidney near the spine, the other in the anterior superior part of the left iliac region. Discontinued all other remedies except the opiates. The issues were each full two inches long by one and a half, and of an oval shape.

June 30th. The issues are discharging freely, no alteration in symptoms.

July 23d. The issues have been open three weeks, and are commencing to heal. The patient is better in every respect. He now seldom rises more than twice or thrice at night to pass water, and much smaller quantities of opium than formerly now suffice to quiet the bowels: appetite and strength improved.

July 25th. Issues healed, no alteration in symptoms since the 23d.

August 6th. Re-opened issues at the request of Mr. H. He declares they have done more for him than all former remedies, and thinks they will effect a cure.

Aug. 25th. The issues have discharged properly for two weeks, and the patient thinks himself nearly free from disease. His strength has so much improved that he is able to perform light labor. The case is evidently doing well.

October 1st. The issues healed about 12th of September, leaving the patient apparently free from disease, except a slightly irritable condition of the rectum, as manifested by tenesmus, but without bloody or mucous discharge. He is now laboring on a farm.

The foregoing cases, which I have abbreviated so far as is consistent with a full understanding of them, seem to me fairly to exhibit the usefulness of the issue used as a means of depletion. The method of applying the nitric acid for the formation of issues is sufficiently simple. All the issues in these cases were made with pure nitric acid in the following manner. A small linen swab, or camel's hair pencil, is procured, with which the acid is painted on the part to be destroyed; continue the application from six to eight minutes, varying with the thickness of the skin and the strength of the caustic. When the cutis becomes rigid from the coagulation of its albumen, so that it can no longer be raised in folds as usual, it is enough: the part is dead down to the sub-cutaneous tissue. It should always be carried thus far: the subsequent ulcer is then never painful. Immediately apply some alkaline solution to prevent further destruction of the parts, which it does by neutralizing the acid. The alkali should be applied for fifteen or twenty minutes; it is well then to apply a warm poultice to facilitate the separation of the eschar, and relieve any remaining inflammation of the part.

I am in the habit of dressing the issue with common adhesive plaster, spread on linen or strong paper. With this simple dressing frequently changed, I have never known a nitric acid issue become either painful or unhealthy.

The foregoing cases and remarks are offered because the utility of *large* issues seems to me not sufficiently regarded by the mass of the profession in this section of our country.

*Aurora, Kane Co., Ill.*, Dec. 26, 1846.

